# Supplementary material for: Predicting Alzheimer’s disease progression using deep recurrent neural networks
Source: Neuroimage. Author manuscript; Available in PMC 2021 Jan 10. (PMC7797176; doi:10.1016/j.neuroimage.2020.117203)
Supplement: 1 [file NIHMS1658212-supplement-1.docx]

# Supplemental Results


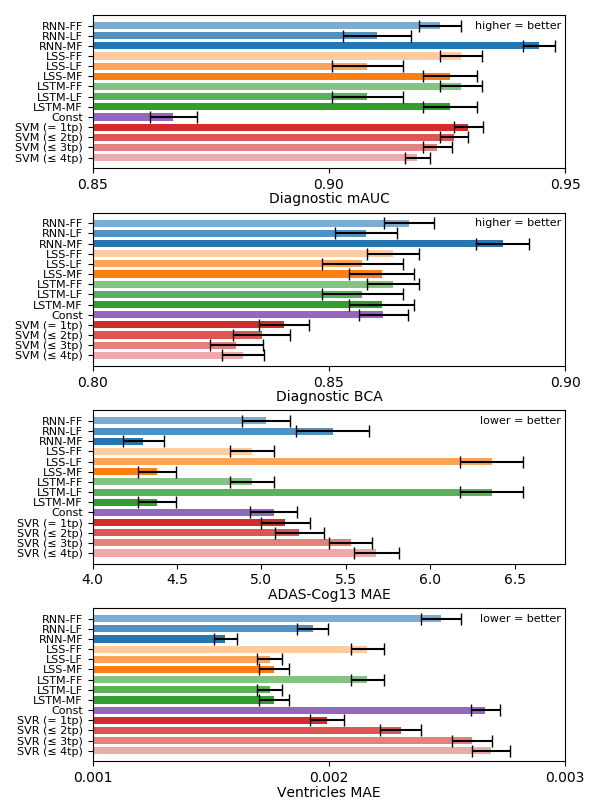


**Figure S1.** Performance of all models averaged across 20 test sets. Error bars show standard error across test sets. For clinical diagnosis, higher mAUC and BCA values indicate better performance. For ADAS-Cog13 and Ventricles, lower MAE indicates better performance. FF indicates forward filling. LF indicates linear filling. MF indicates model filling. SVM/SVR (= 1tp) utilized one input timepoint. SVM/SVR (≤ 2tp) utilized at most 2 input timepoints (see Section 2.5.2 for details) and so on.


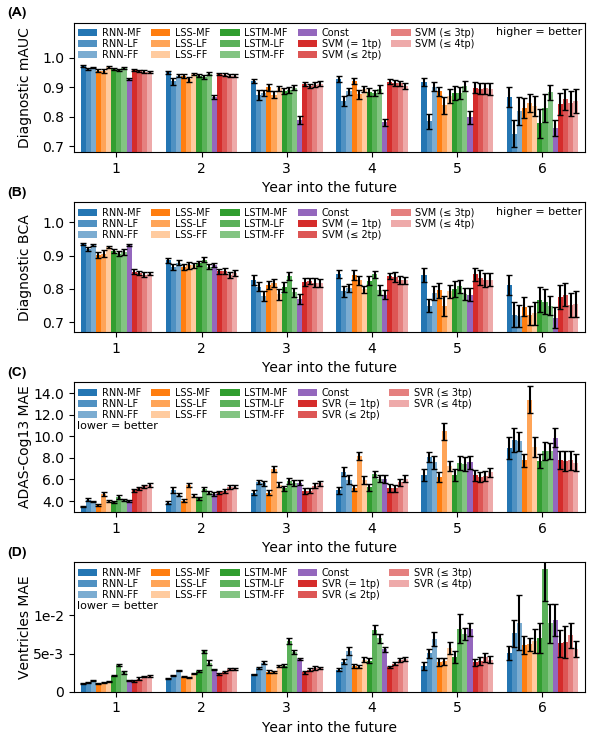


**Figure S2.** Prediction performance from Figure S1 broken down in yearly interval up to 6 years into the future. For clinical diagnosis, higher mAUC and BCA values indicate better performance. For ADAS-Cog13 and Ventricles, lower MAE indicates better performance. FF indicates forward filling. LF indicates linear filling. MF indicates model filling. SVM/SVR (= 1tp) utilized one input timepoint. SVM/SVR (≤ 2tp) utilized at most 2 input timepoints (see Section 2.5.2 for details) and so on.

| Fold No. | Input dropout | Recurrent dropout | Learning rate | Layer size | No. layers | Weight decay |
| --- | --- | --- | --- | --- | --- | --- |
| 1 | 0.5 | 0.5 | 0.006979 | 256 | 3 | 10^-6^ |
| 2 | 0.2 | 0.0 | 0.000316 | 256 | 3 | 10^-7^ |
| 3 | 0.0 | 0.0 | 0.000118 | 128 | 3 | 10^-4^ |
| 4 | 0.2 | 0.0 | 0.000074 | 128 | 3 | 10^-4^ |
| 5 | 0.3 | 0.3 | 0.000682 | 256 | 1 | 10^-6^ |
| 6 | 0.2 | 0.4 | 0.000469 | 256 | 1 | 10^-4^ |
| 7 | 0.4 | 0.0 | 0.000050 | 256 | 3 | 10^-5^ |
| 8 | 0.2 | 0.0 | 0.000118 | 256 | 2 | 10^-4^ |
| 9 | 0.0 | 0.0 | 0.000432 | 256 | 2 | 10^-5^ |
| 10 | 0.0 | 0.0 | 0.000118 | 256 | 2 | 10^-5^ |
| 11 | 0.0 | 0.0 | 0.000118 | 512 | 3 | 10^-4^ |
| 12 | 0.5 | 0.4 | 0.003746 | 256 | 3 | 10^-6^ |
| 13 | 0.1 | 0.1 | 0.000163 | 256 | 2 | 10^-5^ |
| 14 | 0.4 | 0.5 | 0.000649 | 256 | 1 | 10^-7^ |
| 15 | 0.2 | 0.4 | 0.000351 | 128 | 3 | 10^-6^ |
| 16 | 0.5 | 0.3 | 0.006105 | 128 | 3 | 10^-6^ |
| 17 | 0.3 | 0.4 | 0.000703 | 512 | 1 | 10^-7^ |
| 18 | 0.3 | 0.0 | 0.000118 | 128 | 2 | 10^-5^ |
| 19 | 0.1 | 0.2 | 0.000090 | 128 | 3 | 10^-4^ |
| 20 | 0.1 | 0.1 | 0.000224 | 256 | 2 | 10^-5^ |
| mean**±**std | **0.23±0.17** | **0.18±0.20** | **(1.1±2.0) ×10^-3^** | **243±109** | **2.30±0.80** | **(3.3±4.0) ×10^-5^** |

**Table S1.** MinimalRNN hyper-parameters (Forward filling)

| Fold No. | Input dropout | Recurrent dropout | Learning rate | Layer size | No. layers | Weight decay |
| --- | --- | --- | --- | --- | --- | --- |
| 1 | 0.1 | 0.5 | 0.004133 | 128 | 3 | 10^-6^ |
| 2 | 0.2 | 0.0 | 0.000010 | 512 | 3 | 10^-6^ |
| 3 | 0.3 | 0.1 | 0.000073 | 128 | 3 | 10^-6^ |
| 4 | 0.2 | 0.1 | 0.000010 | 512 | 3 | 10^-6^ |
| 5 | 0.1 | 0.0 | 0.000010 | 512 | 3 | 10^-6^ |
| 6 | 0.1 | 0.1 | 0.000010 | 512 | 3 | 10^-5^ |
| 7 | 0.1 | 0.1 | 0.000014 | 512 | 3 | 10^-6^ |
| 8 | 0.1 | 0.0 | 0.000010 | 512 | 3 | 10^-7^ |
| 9 | 0.2 | 0.2 | 0.000044 | 256 | 3 | 10^-5^ |
| 10 | 0.2 | 0.0 | 0.000010 | 512 | 3 | 10^-7^ |
| 11 | 0.5 | 0.3 | 0.006105 | 128 | 3 | 10^-6^ |
| 12 | 0.2 | 0.1 | 0.000030 | 128 | 3 | 10^-5^ |
| 13 | 0.1 | 0.2 | 0.000034 | 256 | 3 | 10^-5^ |
| 14 | 0.2 | 0.1 | 0.000012 | 512 | 3 | 10^-6^ |
| 15 | 0.2 | 0.2 | 0.000029 | 256 | 3 | 10^-6^ |
| 16 | 0.1 | 0.1 | 0.000010 | 512 | 3 | 10^-5^ |
| 17 | 0.5 | 0.1 | 0.000044 | 256 | 3 | 10^-6^ |
| 18 | 0.1 | 0.1 | 0.000088 | 128 | 3 | 10^-5^ |
| 19 | 0.3 | 0.0 | 0.000073 | 128 | 3 | 10^-4^ |
| 20 | 0.0 | 0.5 | 0.003445 | 128 | 3 | 10^-5^ |
| mean**±**std | 0.19±0.13 | 0.14±0.15 | (0.7±1.7) ×10^-3^ | 326±179 | 3.00±0.00 | (0.9±2.2) ×10^-5^ |

**Table S2.** MinimalRNN hyper-parameters (Linear filling)

| Fold No. | Input dropout | Recurrent dropout | Learning rate | Layer size | No. layers | Weight decay |
| --- | --- | --- | --- | --- | --- | --- |
| 1 | 0.1 | 0.4 | 0.00129 | 128 | 2 | 10^-5^ |
| 2 | 0.1 | 0.4 | 0.00133 | 128 | 2 | 10^-7^ |
| 3 | 0.4 | 0.4 | 0.00090 | 128 | 1 | 10^-5^ |
| 4 | 0.1 | 0.4 | 0.00084 | 128 | 2 | 10^-6^ |
| 5 | 0.1 | 0.5 | 0.00084 | 128 | 2 | 10^-5^ |
| 6 | 0.4 | 0.3 | 0.00084 | 128 | 1 | 10^-5^ |
| 7 | 0.0 | 0.2 | 0.00084 | 128 | 2 | 10^-4^ |
| 8 | 0.1 | 0.1 | 0.00094 | 128 | 2 | 10^-7^ |
| 9 | 0.4 | 0.4 | 0.00035 | 128 | 2 | 10^-7^ |
| 10 | 0.1 | 0.3 | 0.00084 | 128 | 2 | 10^-6^ |
| 11 | 0.0 | 0.2 | 0.00084 | 128 | 3 | 10^-6^ |
| 12 | 0.0 | 0.2 | 0.00157 | 128 | 2 | 10^-4^ |
| 13 | 0.0 | 0.5 | 0.00021 | 128 | 2 | 10^-6^ |
| 14 | 0.2 | 0.4 | 0.00147 | 128 | 1 | 10^-7^ |
| 15 | 0.1 | 0.3 | 0.00177 | 128 | 2 | 10^-6^ |
| 16 | 0.1 | 0.4 | 0.00084 | 128 | 2 | 10^-7^ |
| 17 | 0.1 | 0.5 | 0.00084 | 128 | 1 | 10^-4^ |
| 18 | 0.0 | 0.4 | 0.00058 | 128 | 2 | 10^-4^ |
| 19 | 0.2 | 0.5 | 0.00811 | 128 | 2 | 10^-7^ |
| 20 | 0.1 | 0.2 | 0.00130 | 128 | 2 | 10^-7^ |
| mean**±**std | 0.13±0.13 | 0.35±0.12 | (1.3±1.6) ×10^-3^ | 128±0 | 1.85±0.49 | (2.2±4.0) ×10^-7^ |

**Table S3.** MinimalRNN hyper-parameters (Model filling)

| Fold No. | Input dropout | Recurrent dropout | Learning rate | Layer size | No. layers | Weight decay |
| --- | --- | --- | --- | --- | --- | --- |
| 1 | 0.1 | 0.3 | 0.000207 | 128 | 3 | 10^-4^ |
| 2 | 0.0 | 0.0 | 0.000118 | 256 | 2 | 10^-5^ |
| 3 | 0.1 | 0.2 | 0.000316 | 256 | 3 | 10^-7^ |
| 4 | 0.5 | 0.1 | 0.000838 | 128 | 1 | 10^-5^ |
| 5 | 0.2 | 0.3 | 0.000316 | 256 | 1 | 10^-7^ |
| 6 | 0.5 | 0.0 | 0.000118 | 128 | 2 | 10^-5^ |
| 7 | 0.0 | 0.0 | 0.000118 | 256 | 2 | 10^-5^ |
| 8 | 0.2 | 0.1 | 0.000848 | 256 | 3 | 10^-4^ |
| 9 | 0.1 | 0.0 | 0.000062 | 512 | 1 | 10^-7^ |
| 10 | 0.0 | 0.0 | 0.000039 | 512 | 2 | 10^-6^ |
| 11 | 0.4 | 0.2 | 0.000316 | 256 | 1 | 10^-6^ |
| 12 | 0.2 | 0.0 | 0.000848 | 128 | 2 | 10^-4^ |
| 13 | 0.2 | 0.0 | 0.000480 | 512 | 1 | 10^-4^ |
| 14 | 0.2 | 0.2 | 0.000316 | 256 | 1 | 10^-7^ |
| 15 | 0.0 | 0.1 | 0.000118 | 256 | 3 | 10^-7^ |
| 16 | 0.2 | 0.0 | 0.000072 | 128 | 3 | 10^-5^ |
| 17 | 0.3 | 0.0 | 0.000073 | 128 | 2 | 10^-4^ |
| 18 | 0.3 | 0.1 | 0.000054 | 256 | 2 | 10^-5^ |
| 19 | 0.0 | 0.3 | 0.000501 | 128 | 2 | 10^-5^ |
| 20 | 0.0 | 0.0 | 0.000118 | 256 | 2 | 10^-5^ |
| mean**±**std | 0.18±0.16 | 0.10±0.11 | (3.0±2.7) ×10^-4^ | 250±128 | 1.95±0.76 | (2.9±4.2) ×10^-5^ |

**Table S4.** LSS hyper-parameters (Forward filling)

| Fold No. | Input dropout | Recurrent dropout | Learning rate | Layer size | No. layers | Weight decay |
| --- | --- | --- | --- | --- | --- | --- |
| 1 | 0.3 | 0.0 | 0.000011 | 512 | 3 | 10^-4^ |
| 2 | 0.2 | 0.1 | 0.000097 | 128 | 2 | 10^-5^ |
| 3 | 0.1 | 0.1 | 0.002450 | 128 | 2 | 10^-7^ |
| 4 | 0.0 | 0.2 | 0.002242 | 128 | 3 | 10^-7^ |
| 5 | 0.4 | 0.0 | 0.000011 | 512 | 3 | 10^-5^ |
| 6 | 0.5 | 0.2 | 0.000078 | 128 | 3 | 10^-7^ |
| 7 | 0.2 | 0.0 | 0.000011 | 512 | 3 | 10^-7^ |
| 8 | 0.1 | 0.0 | 0.000012 | 512 | 3 | 10^-5^ |
| 9 | 0.1 | 0.1 | 0.000316 | 256 | 1 | 10^-7^ |
| 10 | 0.4 | 0.0 | 0.000010 | 512 | 3 | 10^-6^ |
| 11 | 0.4 | 0.1 | 0.000097 | 128 | 1 | 10^-7^ |
| 12 | 0.3 | 0.1 | 0.000316 | 128 | 1 | 10^-5^ |
| 13 | 0.2 | 0.0 | 0.000084 | 128 | 1 | 10^-7^ |
| 14 | 0.5 | 0.1 | 0.000067 | 128 | 3 | 10^-7^ |
| 15 | 0.2 | 0.1 | 0.000065 | 128 | 2 | 10^-7^ |
| 16 | 0.4 | 0.1 | 0.000096 | 128 | 1 | 10^-6^ |
| 17 | 0.3 | 0.1 | 0.000118 | 128 | 2 | 10^-5^ |
| 18 | 0.2 | 0.1 | 0.004458 | 128 | 2 | 10^-7^ |
| 19 | 0.2 | 0.0 | 0.000011 | 256 | 2 | 10^-7^ |
| 20 | 0.2 | 0.1 | 0.000316 | 128 | 1 | 10^-6^ |
| mean**±**std | 0.26±0.14 | 0.08±0.06 | (0.5±1.2) ×10^-3^ | 237±168 | 2.10±0.85 | (0.8±2.2) ×10^-5^ |

**Table S5.** LSS hyper-parameters (Linear filling)

| Fold No. | Input dropout | Recurrent dropout | Learning rate | Layer size | No. layers | Weight decay |
| --- | --- | --- | --- | --- | --- | --- |
| 1 | 0.4 | 0.1 | 0.00072 | 128 | 2 | 10^-5^ |
| 2 | 0.0 | 0.2 | 0.00056 | 128 | 3 | 10^-6^ |
| 3 | 0.2 | 0.0 | 0.00031 | 512 | 1 | 10^-7^ |
| 4 | 0.2 | 0.1 | 0.00084 | 128 | 2 | 10^-6^ |
| 5 | 0.2 | 0.0 | 0.00031 | 512 | 1 | 10^-7^ |
| 6 | 0.1 | 0.1 | 0.00041 | 256 | 1 | 10^-7^ |
| 7 | 0.1 | 0.0 | 0.00022 | 512 | 1 | 10^-5^ |
| 8 | 0.2 | 0.0 | 0.00031 | 256 | 3 | 10^-7^ |
| 9 | 0.0 | 0.0 | 0.00010 | 256 | 3 | 10^-6^ |
| 10 | 0.2 | 0.2 | 0.00084 | 128 | 2 | 10^-6^ |
| 11 | 0.0 | 0.0 | 0.00011 | 512 | 2 | 10^-4^ |
| 12 | 0.2 | 0.2 | 0.00031 | 256 | 1 | 10^-7^ |
| 13 | 0.1 | 0.2 | 0.00031 | 256 | 1 | 10^-5^ |
| 14 | 0.5 | 0.2 | 0.00066 | 128 | 3 | 10^-7^ |
| 15 | 0.0 | 0.0 | 0.00019 | 256 | 3 | 10^-5^ |
| 16 | 0.0 | 0.2 | 0.00009 | 256 | 3 | 10^-6^ |
| 17 | 0.0 | 0.2 | 0.00031 | 256 | 1 | 10^-5^ |
| 18 | 0.0 | 0.0 | 0.00011 | 256 | 2 | 10^-5^ |
| 19 | 0.2 | 0.5 | 0.00084 | 128 | 2 | 10^-6^ |
| 20 | 0.3 | 0.5 | 0.00084 | 128 | 3 | 10^-4^ |
| mean**±**std | 0.15±0.14 | 0.14±0.15 | (4.3±2.8) ×10^-4^ | 262±140 | 2.00±0.85 | (1.3±3.0) ×10^-5^ |

**Table S6.** LSS hyper-parameters (Model filling)

| Fold No. | Input dropout | Recurrent dropout | Learning rate | Layer size | No. layers | Weight decay |
| --- | --- | --- | --- | --- | --- | --- |
| 1 | 0.0 | 0.0 | 0.000699 | 256 | 1 | 10^-7^ |
| 2 | 0.0 | 0.0 | 0.000035 | 256 | 3 | 10^-5^ |
| 3 | 0.1 | 0.0 | 0.000740 | 128 | 1 | 10^-5^ |
| 4 | 0.2 | 0.1 | 0.000058 | 512 | 2 | 10^-6^ |
| 5 | 0.0 | 0.0 | 0.000721 | 512 | 1 | 10^-7^ |
| 6 | 0.0 | 0.0 | 0.000074 | 512 | 2 | 10^-5^ |
| 7 | 0.1 | 0.2 | 0.001379 | 128 | 2 | 10^-7^ |
| 8 | 0.0 | 0.0 | 0.000175 | 512 | 1 | 10^-7^ |
| 9 | 0.0 | 0.0 | 0.000320 | 512 | 1 | 10^-6^ |
| 10 | 0.0 | 0.0 | 0.000059 | 512 | 1 | 10^-7^ |
| 11 | 0.1 | 0.1 | 0.000096 | 512 | 1 | 10^-4^ |
| 12 | 0.2 | 0.2 | 0.000117 | 256 | 1 | 10^-6^ |
| 13 | 0.1 | 0.0 | 0.000200 | 512 | 2 | 10^-6^ |
| 14 | 0.0 | 0.0 | 0.000373 | 512 | 2 | 10^-7^ |
| 15 | 0.1 | 0.0 | 0.000059 | 512 | 1 | 10^-4^ |
| 16 | 0.0 | 0.0 | 0.000080 | 256 | 2 | 10^-5^ |
| 17 | 0.1 | 0.0 | 0.000643 | 256 | 1 | 10^-6^ |
| 18 | 0.0 | 0.0 | 0.000451 | 512 | 1 | 10^-4^ |
| 19 | 0.0 | 0.0 | 0.000090 | 512 | 1 | 10^-4^ |
| 20 | 0.1 | 0.0 | 0.000432 | 512 | 1 | 10^-6^ |
| mean**±**std | 0.06±0.07 | 0.03±0.07 | (3.4±3.5) ×10^-4^ | 410±147 | 1.40±0.60 | (2.7±4.3) ×10^-5^ |

**Table S7.** LSTM hyper-parameters (Forward filling)

| Fold No. | Input dropout | Recurrent dropout | Learning rate | Layer size | No. layers | Weight decay |
| --- | --- | --- | --- | --- | --- | --- |
| 1 | 0.2 | 0.2 | 0.000208 | 256 | 1 | 10^-7^ |
| 2 | 0.2 | 0.2 | 0.000541 | 128 | 1 | 10^-7^ |
| 3 | 0.2 | 0.2 | 0.000237 | 256 | 1 | 10^-6^ |
| 4 | 0.2 | 0.2 | 0.000058 | 256 | 3 | 10^-7^ |
| 5 | 0.2 | 0.2 | 0.000316 | 256 | 1 | 10^-7^ |
| 6 | 0.2 | 0.2 | 0.000303 | 256 | 1 | 10^-7^ |
| 7 | 0.2 | 0.2 | 0.000130 | 512 | 1 | 10^-6^ |
| 8 | 0.1 | 0.1 | 0.000021 | 512 | 3 | 10^-6^ |
| 9 | 0.2 | 0.2 | 0.000650 | 256 | 1 | 10^-6^ |
| 10 | 0.1 | 0.2 | 0.000074 | 256 | 1 | 10^-7^ |
| 11 | 0.1 | 0.1 | 0.000080 | 256 | 1 | 10^-6^ |
| 12 | 0.2 | 0.2 | 0.000161 | 256 | 1 | 10^-7^ |
| 13 | 0.2 | 0.2 | 0.000237 | 256 | 1 | 10^-6^ |
| 14 | 0.2 | 0.2 | 0.000168 | 256 | 1 | 10^-7^ |
| 15 | 0.2 | 0.2 | 0.000101 | 128 | 3 | 10^-7^ |
| 16 | 0.2 | 0.2 | 0.000072 | 256 | 2 | 10^-7^ |
| 17 | 0.2 | 0.2 | 0.000119 | 256 | 1 | 10^-6^ |
| 18 | 0.2 | 0.2 | 0.000190 | 256 | 1 | 10^-7^ |
| 19 | 0.2 | 0.2 | 0.000191 | 256 | 1 | 10^-7^ |
| 20 | 0.2 | 0.2 | 0.000149 | 256 | 1 | 10^-6^ |
| mean**±**std | 0.19±0.04 | 0.19±0.03 | (2.0±1.6) ×10^-4^ | 269±92 | 1.35±7.45 | (4.6±4.5) ×10^-7^ |

**Table S8.** LSTM hyper-parameters (Linear filling)

| Fold No. | Input dropout | Recurrent dropout | Learning rate | Layer size | No. layers | Weight decay |
| --- | --- | --- | --- | --- | --- | --- |
| 1 | 0.1 | 0.1 | 0.002276 | 512 | 1 | 10^-4^ |
| 2 | 0.0 | 0.1 | 0.010000 | 128 | 1 | 10^-7^ |
| 3 | 0.1 | 0.0 | 0.006868 | 256 | 1 | 10^-4^ |
| 4 | 0.1 | 0.3 | 0.010000 | 512 | 1 | 10^-5^ |
| 5 | 0.1 | 0.4 | 0.008537 | 256 | 1 | 10^-4^ |
| 6 | 0.2 | 0.0 | 0.002276 | 256 | 1 | 10^-5^ |
| 7 | 0.4 | 0.1 | 0.005509 | 256 | 2 | 10^-4^ |
| 8 | 0.1 | 0.4 | 0.010000 | 128 | 2 | 10^-4^ |
| 9 | 0.1 | 0.1 | 0.005038 | 128 | 1 | 10^-7^ |
| 10 | 0.2 | 0.1 | 0.003823 | 128 | 1 | 10^-5^ |
| 11 | 0.1 | 0.4 | 0.010000 | 256 | 1 | 10^-7^ |
| 12 | 0.2 | 0.1 | 0.009419 | 128 | 1 | 10^-4^ |
| 13 | 0.0 | 0.4 | 0.010000 | 128 | 1 | 10^-7^ |
| 14 | 0.2 | 0.1 | 0.010000 | 128 | 1 | 10^-4^ |
| 15 | 0.2 | 0.0 | 0.003699 | 512 | 1 | 10^-7^ |
| 16 | 0.0 | 0.3 | 0.005813 | 128 | 1 | 10^-7^ |
| 17 | 0.2 | 0.0 | 0.004785 | 128 | 1 | 10^-5^ |
| 18 | 0.2 | 0.0 | 0.010000 | 128 | 1 | 10^-7^ |
| 19 | 0.1 | 0.2 | 0.001827 | 128 | 1 | 10^-6^ |
| 20 | 0.1 | 0.1 | 0.006821 | 128 | 1 | 10^-4^ |
| mean**±**std | 0.14±0.09 | 0.16±0.15 | (6.8±3.0) ×10^-3^ | 218±138 | 1.10±0.31 | (4.2±4.9) ×10^-5^ |

**Table S9.** LSTM hyper-parameters (Model filling)

|  | **ADAS-Cog13** | | | | **Ventricles** | | | |
| --- | --- | --- | --- | --- | --- | --- | --- | --- |
| Fold No. | C | Epsilon | Gamma | Kernel | C | Epsilon | Gamma | Kernel |
| 1 | 1.4E-1 | 1.2E-3 | 1.6E-2 | rbf | 1.4E-0 | 1.4E-2 | 2.0E-1 | linear |
| 2 | 3.9E-2 | 8.7E-2 | 1.8E-3 | linear | 8.7E-1 | 7.6E-2 | 1.0E-3 | linear |
| 3 | 1.1E-0 | 2.6E-2 | 1.5E-2 | rbf | 8.4E-1 | 5.7E-2 | 5.1E-1 | linear |
| 4 | 2.3E-0 | 1.1E-3 | 3.4E-3 | rbf | 2.0E-0 | 8.7E-3 | 1.8E-0 | linear |
| 5 | 4.8E-1 | 2.2E-3 | 4.7E-1 | linear | 2.5E-0 | 8.4E-3 | 1.3E-3 | rbf |
| 6 | 4.9E-1 | 6.7E-3 | 1.2E-0 | linear | 7.8E-1 | 1.3E-2 | 7.7E-2 | linear |
| 7 | 1.3E-1 | 2.9E-1 | 2.4E-1 | linear | 1.2E-1 | 1.0E-3 | 2.1E-3 | linear |
| 8 | 1.6E-3 | 1.0E-3 | 4.5E-3 | linear | 2.6E-0 | 2.2E-2 | 1.6E-3 | linear |
| 9 | 7.9E-1 | 2.2E-2 | 6.5E-3 | rbf | 7.8E-1 | 4.9E-3 | 3.4E-1 | linear |
| 10 | 2.9E-1 | 2.6E-3 | 1.4E-2 | rbf | 2.8E-1 | 1.0E-3 | 5.8E-1 | linear |
| 11 | 3.7E-0 | 3.3E-3 | 4.6E-3 | rbf | 6.4E-1 | 3.7E-2 | 4.0E-0 | linear |
| 12 | 3.1E-1 | 1.2E-1 | 4.6E-3 | rbf | 6.7E-1 | 6.5E-2 | 1.2E-2 | linear |
| 13 | 2.3E-2 | 1.6E-2 | 5.9E-3 | linear | 9.3E-0 | 1.6E-2 | 6.9E-3 | rbf |
| 14 | 5.8E-0 | 1.0E-2 | 5.4E-3 | rbf | 1.3E-0 | 3.3E-2 | 5.2E+1 | linear |
| 15 | 3.2E-1 | 9.7E-3 | 1.0E-3 | linear | 2.8E-2 | 3.5E-2 | 3.9E+2 | linear |
| 16 | 4.4E-1 | 5.0E-1 | 1.5E-3 | linear | 1.6E-2 | 1.0E-3 | 1.7E+1 | linear |
| 17 | 4.1E-2 | 1.6E-1 | 2.7E+2 | linear | 3.8E+1 | 1.3E-3 | 2.3E-3 | rbf |
| 18 | 2.4E-1 | 1.2E-2 | 6.1E+1 | linear | 1.3E-2 | 2.3E-2 | 2.6E-3 | linear |
| 19 | 7.8E-3 | 1.0E-2 | 7.6E-1 | linear | 7.4E-2 | 1.0E-3 | 2.3E-3 | linear |
| 20 | 4.8E-1 | 1.0E-3 | 1.0E-3 | linear | 3.0E-1 | 2.1E-3 | 3.7E-1 | linear |
| mean | 8.6E-1 | 6.4E-2 | 1.7E+1 |  | 3.1E-0 | 2.1E-2 | 2.4E+1 |  |
| std | 1.5E-0 | 1.3E-1 | 6.1E+1 |  | 8.4E-0 | 2.3E-2 | 8.8E+1 |  |

**Table S10a.** SVR hyper-parameters (1 input timepoint). Recall that we trained separate SVM/SVR models to predict 10 sets of timepoints (spaced 6 months apart) into the future, i.e., 6, 12, 18, …, 60 months into the future. We note that the hyperparameters are the same across these 10 sets of SVM/SVR models. The reason is to avoid an explosion in the number of hyperparameters.

|  | **Diagnosis** | | | | | | | |
| --- | --- | --- | --- | --- | --- | --- | --- | --- |
| Fold No. | C | Gamma | Kernel |  | Fold No. | C | Gamma | Kernel |
| 1 | 2.9E-2 | 1.0E-2 | linear |  | 11 | 4.2E-3 | 1.3E-2 | linear |
| 2 | 3.9E-3 | 3.6E-3 | linear |  | 12 | 1.2E-1 | 1.2E-0 | linear |
| 3 | 2.6E-3 | 8.5E-3 | linear |  | 13 | 6.6E-3 | 2.0E-1 | linear |
| 4 | 6.3E-3 | 2.1E-3 | linear |  | 14 | 4.2E-3 | 2.5E-2 | linear |
| 5 | 1.5E-2 | 2.4E-3 | linear |  | 15 | 5.5E-3 | 1.2E-3 | linear |
| 6 | 5.9E-3 | 2.2E-3 | linear |  | 16 | 2.1E-2 | 7.2E+1 | linear |
| 7 | 3.3E-3 | 1.1E-2 | linear |  | 17 | 8.1E-2 | 2.6E-2 | linear |
| 8 | 4.4E-1 | 4.8E-3 | linear |  | 18 | 8.9E-1 | 2.8E-3 | linear |
| 9 | 7.3E-1 | 1.7E-2 | rbf |  | 19 | 8.5E-2 | 2.9E-2 | linear |
| 10 | 7.4E-3 | 1.2E-3 | linear |  | 20 | 6.0E-3 | 7.8E-0 | linear |
|  |  |  |  |  | mean | 1.2E-01 | 4.1E+0 |  |
|  |  |  |  |  | Std | 2.6E-01 | 1.6E+1 |  |

**Table S10b.** SVM hyper-parameters (1 input timepoint). Recall that we trained separate SVM/SVR models to predict 10 sets of timepoints (spaced 6 months apart) into the future, i.e., 6, 12, 18, …, 60 months into the future. We note that the hyperparameters are the same across these 10 sets of SVM/SVR models. The reason is to avoid an explosion in the number of hyperparameters.

|  | **ADAS-Cog13** | | | | **Ventricles** | | | |
| --- | --- | --- | --- | --- | --- | --- | --- | --- |
| Fold No. | C | Epsilon | Gamma | Kernel | C | Epsilon | Gamma | Kernel |
| 1 | 1.3E-0 | 1.0E-3 | 2.9E-3 | rbf | 4.7E-2 | 8.2E-2 | 1.4E-2 | linear |
| 2 | 3.2E-3 | 1.3E-3 | 4.5E+1 | linear | 1.1E-2 | 1.5E-3 | 3.4E-0 | linear |
| 3 | 2.1E-0 | 1.6E-3 | 4.2E-3 | rbf | 5.8E-1 | 1.6E-1 | 2.5E-1 | linear |
| 4 | 2.4E-1 | 4.1E-3 | 4.2E-3 | rbf | 1.8E-1 | 1.2E-1 | 1.2E-1 | linear |
| 5 | 2.9E-3 | 1.0E-3 | 1.8E-3 | linear | 3.9E-2 | 5.3E-2 | 2.4E-2 | linear |
| 6 | 2.6E-1 | 5.1E-3 | 7.7E-3 | rbf | 2.4E-1 | 2.3E-1 | 4.3E-1 | linear |
| 7 | 4.1E-1 | 3.8E-1 | 1.0E-3 | linear | 1.2E-1 | 1.1E-1 | 1.0E-3 | linear |
| 8 | 7.6E-1 | 3.8E-3 | 2.9E-3 | rbf | 6.7E-0 | 2.5E-2 | 9.9E+1 | linear |
| 9 | 8.2E-2 | 9.1E-2 | 4.0E-3 | linear | 1.9E-2 | 1.8E-2 | 9.0E-3 | linear |
| 10 | 1.5E-1 | 3.9E-3 | 9.8E-3 | rbf | 4.2E-2 | 6.5E-2 | 7.6E-3 | linear |
| 11 | 2.5E-0 | 5.5E-2 | 6.6E+1 | linear | 2.7E-2 | 1.5E-3 | 1.8E-0 | linear |
| 12 | 2.4E-3 | 1.6E-2 | 2.8E-0 | linear | 1.0E-2 | 1.1E-3 | 1.1E-1 | linear |
| 13 | 3.8E-1 | 5.6E-3 | 3.3E-3 | rbf | 2.4E-0 | 1.6E-1 | 1.2E-0 | linear |
| 14 | 2.4E-1 | 2.6E-3 | 4.2E-3 | rbf | 8.6E-0 | 2.1E-1 | 1.6E+1 | linear |
| 15 | 1.0E-3 | 4.4E-3 | 1.1E-1 | linear | 2.0E-2 | 1.0E-3 | 1.1E+1 | linear |
| 16 | 2.4E-1 | 3.7E-3 | 1.1E-2 | rbf | 7.8E-0 | 2.2E-1 | 6.2E-2 | linear |
| 17 | 1.5E-1 | 1.5E-1 | 1.0E-2 | linear | 1.5E+1 | 4.0E-2 | 8.6E-1 | linear |
| 18 | 1.7E-3 | 6.8E-2 | 2.8E-0 | linear | 2.0E-2 | 1.0E-3 | 1.2E-0 | linear |
| 19 | 7.4E-1 | 3.5E-3 | 1.1E-3 | rbf | 6.2E-2 | 1.1E-1 | 7.3E-1 | linear |
| 20 | 2.4E-1 | 2.9E-2 | 6.7E-3 | rbf | 6.0E-1 | 1.9E-1 | 5.8E-1 | linear |
| mean | 4.9E-1 | 4.1E-2 | 5.9E+0 |  | 2.1E-0 | 9.0E-2 | 6.9E+0 |  |
| std | 7.1E-1 | 8.8E-2 | 1.7E+1 |  | 4.0E-0 | 8.1E-2 | 2.2E+1 |  |

**Table S11a.** SVR hyper-parameters (2 input timepoints). Recall that we trained separate SVM/SVR models to predict 10 sets of timepoints (spaced 6 months apart) into the future, i.e., 6, 12, 18, …, 60 months into the future. We note that the hyperparameters are the same across these 10 sets of SVM/SVR models. The reason is to avoid an explosion in the number of hyperparameters.

|  | **Diagnosis** | | | | | | | |
| --- | --- | --- | --- | --- | --- | --- | --- | --- |
| Fold No. | C | Gamma | Kernel |  | Fold No. | C | Gamma | Kernel |
| 1 | 4.5E-1 | 3.3E+2 | linear |  | 11 | 5.4E-3 | 1.5E-3 | linear |
| 2 | 1.0E-3 | 2.3E-2 | linear |  | 12 | 2.4E-1 | 6.8E-3 | linear |
| 3 | 4.6E-3 | 2.8E-1 | linear |  | 13 | 9.6E-3 | 6.3E-1 | linear |
| 4 | 2.4E-3 | 1.3E-1 | linear |  | 14 | 2.4E-3 | 6.7E-3 | linear |
| 5 | 1.8E-2 | 1.1E-1 | linear |  | 15 | 2.2E-2 | 2.2E+2 | linear |
| 6 | 1.4E-1 | 3.6E-2 | linear |  | 16 | 1.1E-1 | 2.8E-3 | linear |
| 7 | 1.9E-2 | 3.9E-2 | linear |  | 17 | 5.4E-2 | 1.1E+2 | linear |
| 8 | 2.8E-2 | 7.1E-2 | linear |  | 18 | 1.9E-1 | 6.5E-0 | linear |
| 9 | 3.6E-2 | 5.5E+1 | linear |  | 19 | 8.7E-2 | 5.1E-3 | linear |
| 10 | 3.5E-3 | 2.0E-1 | linear |  | 20 | 4.4E-3 | 7.2E-2 | linear |
|  |  |  |  |  | mean | 7.2E-2 | 3.6E+1 |  |
|  |  |  |  |  | std | 1.1E-1 | 8.8E+1 |  |

**Table S11b.** SVM hyper-parameters (2 input timepoints). Recall that we trained separate SVM/SVR models to predict 10 sets of timepoints (spaced 6 months apart) into the future, i.e., 6, 12, 18, …, 60 months into the future. We note that the hyperparameters are the same across these 10 sets of SVM/SVR models. The reason is to avoid an explosion in the number of hyperparameters.

|  | **ADAS-Cog13** | | | | **Ventricles** | | | |
| --- | --- | --- | --- | --- | --- | --- | --- | --- |
| Fold No. | C | Epsilon | Gamma | Kernel | C | Epsilon | Gamma | Kernel |
| 1 | 2.3E-2 | 5.4E-1 | 2.2E-3 | linear | 1.2E-2 | 2.5E-3 | 1.1E-2 | linear |
| 2 | 3.3E-3 | 1.1E-1 | 1.0E-3 | linear | 1.1E-2 | 1.0E-3 | 1.4E-0 | linear |
| 3 | 2.9E-3 | 1.6E-3 | 3.8E+1 | linear | 1.7E-2 | 3.3E-2 | 1.8E-2 | linear |
| 4 | 8.0E-0 | 2.5E-1 | 1.0E-3 | rbf | 4.9E-2 | 9.1E-2 | 7.4E-3 | linear |
| 5 | 1.5E-3 | 1.0E-3 | 5.0E+1 | linear | 6.3E+1 | 8.9E-2 | 1.0E-3 | rbf |
| 6 | 6.5E-2 | 4.2E-3 | 9.4E-3 | rbf | 3.5E-2 | 8.1E-2 | 1.8E-2 | linear |
| 7 | 9.1E-2 | 5.7E-1 | 1.1E-2 | linear | 4.0E-2 | 3.4E-2 | 1.4E+1 | linear |
| 8 | 4.5E-1 | 1.0E-2 | 4.2E-3 | rbf | 5.0E-0 | 3.2E-3 | 2.2E-3 | rbf |
| 9 | 2.1E-0 | 6.8E-3 | 1.2E-3 | rbf | 5.6E-1 | 2.2E-1 | 1.1E+1 | linear |
| 10 | 3.0E-1 | 2.6E-3 | 3.6E-3 | rbf | 1.5E-2 | 1.9E-2 | 1.5E-3 | linear |
| 11 | 3.4E-2 | 1.5E-1 | 1.4E-0 | linear | 4.1E-2 | 1.3E-3 | 3.9E-2 | linear |
| 12 | 2.6E-3 | 6.4E-3 | 1.9E-3 | linear | 1.2E+1 | 5.0E-2 | 1.0E-3 | rbf |
| 13 | 1.6E-1 | 4.1E-1 | 1.1E-2 | linear | 1.0E-2 | 1.0E-3 | 1.2E-2 | linear |
| 14 | 5.1E-2 | 6.3E-2 | 1.7E-2 | linear | 1.1E-1 | 9.1E-2 | 3.0E-1 | linear |
| 15 | 2.5E-2 | 2.9E-1 | 1.5E-1 | linear | 3.6E-2 | 6.7E-2 | 3.4E-3 | linear |
| 16 | 2.3E-1 | 5.6E-3 | 8.3E-3 | rbf | 1.8E+1 | 3.0E-2 | 4.2E+1 | linear |
| 17 | 3.2E-2 | 8.5E-2 | 1.4E-2 | linear | 7.1E-2 | 9.6E-2 | 3.0E-3 | linear |
| 18 | 3.2E-1 | 4.3E-3 | 3.0E-3 | rbf | 2.9E-2 | 6.0E-2 | 3.3E-3 | linear |
| 19 | 1.8E-1 | 5.1E-3 | 4.9E-3 | rbf | 2.8E-1 | 2.1E-1 | 7.1E-2 | linear |
| 20 | 2.4E-1 | 3.7E-3 | 4.2E-3 | rbf | 2.4E+1 | 1.4E-1 | 7.5E-2 | linear |
| mean | 6.1E-1 | 1.3E-1 | 4.5E+0 |  | 6.2E+0 | 6.6E-2 | 3.5E-0 |  |
| std | 1.8E-0 | 1.9E-1 | 1.4E+1 |  | 1.5E+1 | 6.5E-2 | 9.9E-0 |  |

**Table 12a.** SVR hyper-parameters (3 input timepoints). Recall that we trained separate SVM/SVR models to predict 10 sets of timepoints (spaced 6 months apart) into the future, i.e., 6, 12, 18, …, 60 months into the future. We note that the hyperparameters are the same across these 10 sets of SVM/SVR models. The reason is to avoid an explosion in the number of hyperparameters.

|  | **Diagnosis** | | | | | | | |
| --- | --- | --- | --- | --- | --- | --- | --- | --- |
| Fold No. | C | Gamma | Kernel |  | Fold No. | C | Gamma | Kernel |
| 1 | 1.1E-1 | 1.3E+0 | linear |  | 11 | 4.5E-2 | 1.0E+1 | linear |
| 2 | 4.2E-3 | 5.3E+2 | linear |  | 12 | 5.2E-2 | 4.4E-1 | linear |
| 3 | 4.6E-2 | 5.4E+0 | linear |  | 13 | 2.9E-2 | 2.8E+1 | linear |
| 4 | 5.2E-3 | 7.0E+1 | linear |  | 14 | 2.0E-2 | 1.3E-3 | linear |
| 5 | 3.0E-2 | 3.6E+2 | linear |  | 15 | 7.7E-2 | 1.3E-1 | linear |
| 6 | 4.5E-2 | 1.2E-2 | linear |  | 16 | 3.3E-2 | 4.5E-1 | linear |
| 7 | 2.1E-3 | 1.0E-3 | linear |  | 17 | 6.3E-2 | 6.1E-0 | linear |
| 8 | 4.1E-2 | 2.7E-2 | linear |  | 18 | 1.3E-2 | 6.8E-2 | linear |
| 9 | 2.3E-2 | 3.5E-2 | linear |  | 19 | 8.1E-3 | 9.3E-0 | linear |
| 10 | 5.9E-2 | 5.8E-1 | linear |  | 20 | 8.4E-3 | 1.2E-2 | linear |
|  |  |  |  |  | mean | 3.6E-2 | 5.1E+1 |  |
|  |  |  |  |  | std | 2.7E-2 | 1.4E+2 |  |

**Table 12b.** SVM hyper-parameters (3 input timepoints). Recall that we trained separate SVM/SVR models to predict 10 sets of timepoints (spaced 6 months apart) into the future, i.e., 6, 12, 18, …, 60 months into the future. We note that the hyperparameters are the same across these 10 sets of SVM/SVR models. The reason is to avoid an explosion in the number of hyperparameters.

|  | **ADAS-Cog13** | | | | **Ventricles** | | | |
| --- | --- | --- | --- | --- | --- | --- | --- | --- |
| Fold No. | C | Epsilon | Gamma | Kernel | C | Epsilon | Gamma | Kernel |
| 1 | 6.1E-2 | 6.3E-1 | 8.1E-3 | linear | 3.9E+1 | 2.5E-2 | 1.0E-3 | rbf |
| 2 | 2.0E-2 | 1.7E-1 | 6.9E-2 | linear | 1.7E-2 | 1.5E-2 | 4.0E-3 | linear |
| 3 | 1.3E-2 | 8.7E-2 | 7.3E-2 | linear | 2.0E-2 | 1.9E-2 | 1.0E-1 | linear |
| 4 | 2.5E-3 | 1.6E-3 | 9.2E-3 | linear | 2.2E-2 | 2.8E-2 | 3.3E-3 | linear |
| 5 | 3.6E-3 | 1.6E-1 | 4.6E-3 | linear | 2.2E-2 | 1.0E-3 | 8.5E-2 | linear |
| 6 | 1.7E-1 | 1.6E-3 | 6.7E-3 | rbf | 2.2E-2 | 4.0E-2 | 2.7E-3 | linear |
| 7 | 3.6E-2 | 6.8E-1 | 5.8E-3 | linear | 2.4E-2 | 1.0E-3 | 1.0E-3 | linear |
| 8 | 4.1E-1 | 1.2E-3 | 2.7E-3 | rbf | 4.5E-2 | 1.5E-2 | 4.5E+0 | linear |
| 9 | 2.2E-2 | 5.2E-1 | 2.3E-2 | linear | 2.9E-2 | 2.3E-2 | 7.3E-1 | linear |
| 10 | 6.0E-3 | 2.0E-3 | 1.0E-3 | linear | 2.4E-2 | 5.8E-2 | 2.8E-1 | linear |
| 11 | 1.5E-2 | 1.2E-2 | 2.2E+1 | linear | 4.6E-2 | 2.5E-2 | 1.0E-3 | linear |
| 12 | 1.3E-2 | 3.7E-1 | 2.8E-1 | linear | 5.7E-2 | 1.0E-1 | 1.5E+2 | linear |
| 13 | 2.2E-2 | 4.3E-1 | 2.6E-2 | linear | 1.9E-2 | 1.3E-3 | 1.3E-2 | linear |
| 14 | 4.0E-2 | 5.3E-1 | 2.3E-0 | linear | 1.4E-1 | 1.2E-1 | 2.1E+1 | linear |
| 15 | 1.1E-2 | 9.6E-3 | 6.7E-1 | linear | 1.6E-2 | 1.5E-3 | 1.8E-1 | linear |
| 16 | 1.7E-1 | 1.0E-3 | 5.2E-3 | rbf | 4.0E-2 | 5.6E-2 | 7.3E-1 | linear |
| 17 | 2.9E-2 | 4.7E-1 | 1.6E-2 | linear | 1.2E-2 | 1.0E-3 | 8.0E-3 | linear |
| 18 | 4.1E-3 | 1.9E-2 | 1.4E-3 | linear | 1.7E-2 | 1.0E-3 | 4.4E-2 | linear |
| 19 | 2.2E-1 | 3.7E-3 | 4.2E-3 | rbf | 3.5E+1 | 4.3E-2 | 8.3E-1 | linear |
| 20 | 3.2E-1 | 2.9E-3 | 3.3E-3 | rbf | 6.0E-2 | 7.3E-2 | 6.7E-1 | linear |
| mean | 8.0E-2 | 2.1E-1 | 1.3E-0 |  | 3.7E+0 | 3.2E-2 | 8.8E+0 |  |
| std | 1.2E-1 | 2.5E-1 | 4.8E-0 |  | 1.1E+1 | 3.4E-2 | 3.3E+1 |  |

**Table 13a.** SVR hyper-parameters (4 input timepoints). Recall that we trained separate SVM/SVR models to predict 10 sets of timepoints (spaced 6 months apart) into the future, i.e., 6, 12, 18, …, 60 months into the future. We note that the hyperparameters are the same across these 10 sets of SVM/SVR models. The reason is to avoid an explosion in the number of hyperparameters.

|  | **Diagnosis** | | | | | | | |
| --- | --- | --- | --- | --- | --- | --- | --- | --- |
| Fold No. | C | Gamma | Kernel |  | Fold No. | C | Gamma | Kernel |
| 1 | 9.2E-2 | 1.2E+0 | linear |  | 11 | 3.2E-2 | 5.2E-1 | linear |
| 2 | 8.1E-2 | 1.2E+1 | linear |  | 12 | 4.9E-2 | 1.0E+3 | linear |
| 3 | 7.0E-2 | 4.1E-3 | linear |  | 13 | 1.3E-2 | 2.3E-2 | linear |
| 4 | 1.4E-2 | 2.0E+1 | linear |  | 14 | 2.2E-2 | 1.4E-3 | linear |
| 5 | 4.4E-2 | 1.0E-3 | linear |  | 15 | 2.7E-2 | 3.9E-3 | linear |
| 6 | 2.4E-2 | 4.8E+1 | linear |  | 16 | 2.9E-2 | 1.3E+1 | linear |
| 7 | 9.4E-3 | 4.8E+0 | linear |  | 17 | 7.1E-2 | 1.3E+1 | linear |
| 8 | 2.5E-2 | 1.5E-1 | linear |  | 18 | 1.1E-1 | 6.1E-2 | linear |
| 9 | 2.2E-2 | 6.6E-3 | linear |  | 19 | 3.5E-2 | 4.2E-2 | linear |
| 10 | 6.4E-3 | 1.0E+3 | linear |  | 20 | 2.9E-2 | 5.7E-1 | linear |
|  |  |  |  |  | mean | 4.0E-2 | 1.1E+2 |  |
|  |  |  |  |  | std | 2.9E-2 | 3.1E+2 |  |

**Table 13b.** SVM hyper-parameters (4 input timepoints). Recall that we trained separate SVM/SVR models to predict 10 sets of timepoints (spaced 6 months apart) into the future, i.e., 6, 12, 18, …, 60 months into the future. We note that the hyperparameters are the same across these 10 sets of SVM/SVR models. The reason is to avoid an explosion in the number of hyperparameters.

|  | mAUC (more=better) | BCA (more=better) | ADAS-Cog13 (less=better) | Ventricles (less=better) |
| --- | --- | --- | --- | --- |
| RNN–MF | **0.944** ± 0.014 | **0.887** ± 0.024 | **4.30** ± 0.53 | **0.00156** ± 0.00022 |
| SVM/SVR (= 1tp)  (MFPCA) | 0.927 ± 0.013  (p = 0.011) | 0.837 ± 0.023  (p = 2.5×10^-7^) | 5.19 ± 0.62  (p = 1.8×10^-4^) | 0.00203 ± 0.00031  (p = 7.3×10^-5^) |
| SVM/SVR (≤ 2tp)  (MFPCA) | 0.925 ± 0.013  (p = 0.002) | 0.834 ± 0.026  (p = 2.8×10^-6^) | 5.22 ± 0.63  (p = 1.1×10^-4^) | 0.00278 ± 0.00037  (p = 2.7×10^-7^) |
| SVM/SVR (≤ 3tp)  (MFPCA) | 0.921 ± 0.013  (p = 0.001) | 0.824 ± 0.025  (p = 2.6×10^-7^) | 5.46 ± 0.55  (p = 4.5×10^-7^) | 0.00377 ± 0.00037  (p = 5.9×10^-7^) |
| SVM/SVR (≤ 4tp)  (MFPCA) | 0.918 ± 0.012  (p = 2.2×10^-5^) | 0.826 ± 0.019  (p = 4.1×10^-7^) | 5.62 ± 0.58  (p = 9.4×10^-7^) | 0.00436 ± 0.00035  (p = 1.2×10^-9^) |
| SVM/SVR (= 1tp)  (Linear) | 0.929 ± 0.013  (p = 0.011) | 0.841 ± 0.023  (p = 2.5×10^-7^) | 5.14 ± 0.62  (p = 1.8×10^-4^) | 0.00199 ± 0.00031  (p = 7.3×10^-5^) |
| SVM/SVR (≤ 2tp)  (Linear) | 0.926 ± 0.013  (p = 0.002) | 0.836 ± 0.026  (p = 2.8×10^-6^) | 5.23 ± 0.63  (p = 1.1×10^-4^) | 0.00230 ± 0.00037  (p = 2.7×10^-7^) |
| SVM/SVR (≤ 3tp)  (Linear) | 0.923 ± 0.013  (p = 0.001) | 0.830 ± 0.025  (p = 2.6×10^-7^) | 5.53 ± 0.55  (p = 4.5×10^-7^) | 0.00261 ± 0.00037  (p = 5.9×10^-7^) |
| SVM/SVR (≤ 4tp)  (Linear) | 0.919 ± 0.012  (p = 2.2×10^-5^) | 0.832 ± 0.019  (p = 4.1×10^-7^) | 5.68 ± 0.58  (p = 9.4×10^-7^) | 0.00269 ± 0.00035  (p = 1.2×10^-9^) |

**Table S14.** Comparison of minimalRNN with model filling strategy (RNN-MF) and SVM/SVR models with MFPCA. We observe that there was little performance difference between using MPFCA and linear interpolation (Table 4) for filling in missing data for the SVM/SVR models.
